# Job Demands, Engagement, and Turnover Intentions in Polish Nurses: The Role of Work-Family Interface

**DOI:** 10.3389/fpsyg.2016.01621

**Published:** 2016-11-01

**Authors:** Anna M. Dåderman, Beata A. Basinska

**Affiliations:** ^1^Division of Psychology, Education and Organisational Studies, Department of Social and Behavioural Studies, University West, TrollhättanSweden; ^2^Faculty of Management and Economics, Gdansk University of Technology, GdanskPoland; ^3^Faculty of Psychology, University of Social Sciences and Humanities, WarsawPoland

**Keywords:** work-family conflict, family work conflict, interpersonal conflicts, workload, work engagement, turnover intentions

## Abstract

**Background:** Poland has lower ratios of employed registered nurses per 1,000 inhabitants than the EU average. Polish nurses work under miserable conditions without assisting personnel, and they reconcile their professional demands with responsibilities for their families; 96% of them are women.

**Rationale/Aims:** This study uses Hobfoll’s conservation of resources (CORs) theory to explain the role of various resources in the improvement of work conditions in the nursing profession. Work-family conflict (WFC) and family work conflict (FWC) threaten to deplete nurses’ resources. This paper set out to (1) examine the extent to which perceived job demands (workload and interpersonal conflicts at work) and engagement (vigor, dedication, and absorption) are associated with turnover intentions (the intention to leave the present workplace and the intention to leave the nursing profession); (2) attempt to determine whether levels of WFC and FWC moderate these associations.

**Design/Method:** This study comprised 188 female registered nurses. The inclusion criterion was to live with a partner and/or have children.

**Results:** WFC was moderately related to FWC. Hierarchical regression analyses showed that only high job demands and low vigor were significantly associated with turnover intentions. WFC was experienced more intensively than FWC. Job demands, vigor, dedication, and turnover intentions had a strong effect on WFC, while absorption had a strong effect on FWC. However, levels of WFC and FWC did not significantly moderate these associations.

**Originality/Conclusion:** The study produces new knowledge by examining a constellation of job demands, work engagement and WFC, which reflect the management of personal resources. Results from such a constellation in nurses from countries with a post-transformational economic system have not previously been discussed in the light of COR theory. Most importantly, we conclude that WFC does not intensify turnover intentions.

## Introduction

Nursing work is demanding and stressful, which is often associated with poor well-being (e.g., Sorgaard et al., 2010; Pisanti et al., 2011, 2015; Purcell et al., 2011; Giorgi et al., 2016). Most nurses are women (in Poland, 96%) who traditionally must reconcile professional demands with responsibilities for the family. In recent years, the demands on this profession have increased, while the reward have decreased, which has resulted in miserable work conditions, and consequently in turnover intentions. Heavy workload, low salaries, as well as lack of recognition and prestige, are huge problems for nurses in post-transformation countries. Nursing is an alarmingly languishing profession in Poland. Due to a lack of nurses, patient safety is seriously threatened. At home, nurses worry not only about whether they have hurt their patients’ feelings, but also whether they have committed malpractice. In one Polish hospital, nurses took the dramatic decision to start a protest against the poor working conditions (24th May 2016).

### The Social and Cultural Context

Economic transition and rapid development have transformed the demands on employees in Poland. The most important changes concern the commercialisation of medical institutions and the introduction of work for hire in place of contracts of employment for medical staff (Strózik, 2006). A relatively low salary and the low prestige of the nursing profession co-occur with work overload, extended working hours and job insecurity (Basinska and Wilczek-Ruzyczka, 2011, 2013; Borowiak et al., 2011). The number of nurses who abandon the profession has increased in recent years (Main Chamber of Nurses and Midwives, 2010; National Trade Union of Nurses and Midwives, 2015) and others have left the country to work in other EU member states (Binkowska-Bury et al., 2010; Beckford and Macfarlane, 2014). Further, few young women are interested in becoming nurses (Borowiak et al., 2011). The mean age of Polish nurses is 45 years. Poland has a lower number of employed nurses per 1,000 inhabitants than the average in the EU (5.2, to be compared to 10.2 in Sweden and 14.8 in Norway; Eurostat Statistics Explained, 2015). Moreover, Polish registered nurses are not supported by nursing associate professionals or health care assistants. In the light of the need to retain nurses in Poland it may prove valuable to explore how the nurses manage their personal resources, as well as the relationship between turnover intentions and work-related variables.

A system based on dual earners, in which females are subject to a double burden, is adopted in post-transformation countries. Additional demands on women in these countries arise from a tradition in which women take care of elderly parents, and therefore must play several roles: mother, wife, and caring daughter.

### Theoretical Framework: Hobfoll’s Theory of the Conservation of Resources (COR)

Hobfoll’s COR theory (Hobfoll, 1989, 2011, 2012) was applied as a theoretical framework for this study. COR theory states that people strive to retain, gain and protect their resources, such as health, well-being, family, money, time, or energy (e.g., knowledge, credit). These resources are universally valued. The theory states that stress occurs when there is a loss of resources, or a threat of loss. People attempt to preserve, retain, protect, and create new resources in order to maximize their ability to manage and mitigate the stress.

Conservation of resources theory is built on four principles (Hobfoll, 1989, 2011, 2012). The first principle states that in comparison to resource gain, “resource loss is disproportionate in terms of not only degree, but also speed” (Hobfoll, 2011, p. 117). The second principle states that people “must invest resources in order to protect against resource loss, recover from losses and gain resources” (Hobfoll, 2011, p. 117), which means that those with fewer resources are more sensitive to losses than to gains and pay more to limit a loss than to achieve a gain. The third and fourth principles state that, as regards resources, “gain and loss cycles, respectively, occur in chronically stressful conditions, or where individuals or organizations are resource poor and any major stressor occurs” (Hobfoll, 2011, p. 118).

Hobfoll (1989, 2011, 2012) uses metaphors to explain different concepts and the relationships between resources. For example, a development of resources may be visualized as a camel caravan in which resources support each other and their additive actions are worth more than a single resource. The concept of “loss spirals” (Hobfoll, 1989) is often used to explain why people with a low level of resources are more vulnerable to a loss of resources and less able to regain them, and are likely to take a defensive position to protect their resources, at least when under high stress. Thus, loss spirals develop because these people “lack the resources to offset loss. If resources are used to prevent loss of other resources, such loss would be predicted to lead to further decreases in the likelihood of possessing necessary resource reserves” (p. 519).

### High Work-Family Conflict (WFC) and Family Work Conflict (FWC) as a Threat of Loss of Resources

The conflict between professional and family life is bilateral (Grzywacz and Marks, 2000): consequences of excessive job demands can be transferred into family life and *vice versa*; troubles at home can be transferred to work. We have adopted the terms work-family conflict (WFC) and family work conflict (FWC) from Netemeyer et al. (1996) who stated that “WFC is a form of inter-role conflict in which the general demands of, time devoted to, and strain created by the job interfere with performing family related responsibilities. FWC is a form of inter-role conflict in which the general demands of, time devoted to, and strain created by the family interfere with performing work-related responsibilities” (p. 401). According to Netemeyer et al. (1996) WFC seriously impairs the employee’s performance. WFC may lead to a loss of resources in the process of seeking a compromise between work and family roles (Grandey and Cropanzano, 1999). In particular, when work is performed under miserable conditions and still involves the responsibility for other persons’ safety, employees with high WFC may deplete their personal resources such as energy, motivation and engagement. Excessive demands in the work domain may reduce the available resources and impair function in the family domain.

According to COR theory, high quantitative and interpersonal job demands lead to depletion of resources, mainly energetic and emotional resources, because demands require effort and costs, mainly in the form of physical, mental, and emotional energy. Next, an imbalance between work and private life can pose a threat to various resources. WFC means that the nurse’s work uses up too many resources, which limits the use of these resources at home. Too much FWC means that the nurse’s home requires more resources than are available, and that this situation impedes her work. Further, work engagement is related to motivation to invest one’s resources as well as to retain and build resources. Engagement, as a resource, is the internal energy that is invested in the nurse’s work. Finally, intentions of turnover, that is, of seeking a new job, can be an attempt to protect resources and prevent further losses. In conclusion, job demands, conflicts between work and family, work engagement, and turnover intentions are closely related to the management of personal resources. COR theory has been supported by past research on WFC and FWC (Grandey and Cropanzano, 1999; Netemeyer et al., 2005; Nohe et al., 2015; Lee et al., 2016).

### Miserable Job Demands (Quantitative Workload and Interpersonal Conflicts at Work) Deplete Resources

Job demands may be experienced as important stressors that interfere with family life. We examine two types of nurses’ job demands: those concerning the tasks to be carried out (quantitative workload, that is, too much work to do in too short time), and interpersonal relations (possible conflicts with patients, patients’ families, and other medical staff).

Quantitative workload is defined as “the sheer volume of work required of an employee” (Spector and Jex, 1998, p. 358). Shaffer et al. (2011) have shown that job demands include broad categories of working hours and time pressure, work expectations and role stressors. Quantitative workload is correlated with role conflict and frustration (Spector and Jex), fatigue after the working day (Basinska and Wilczek-Ruzyczka, 2011) and burnout (Pisanti et al., 2011). A high workload is related to a certain level of uncertainty (feelings of worry and anxiety; Beehr and Bhagat, 1985), because an employee who has too much to do may neglect some aspects of work life or family life. The low ratio of nurses/patients leads to a high workload, which may result in excessive absorption, a difficulty to detach oneself from work. For example, if a nurse has too much work to do with her patients, she has to use extra resources in the form of emotional energy. If she has no such extra resources, and, in addition, has no assistants and too few co-workers, her resources are depleted.

Interpersonal conflicts at the workplace are one of the most important stressors (Keenan and Newton, 1985); for instance, the risk that nurses are exposed to violence is significant (Nachreiner et al., 2005; Happell, 2008). Conflicts at work are associated with lower efficiency of the team and lower productivity (Alper et al., 2000; Dunlop and Lee, 2004). Interpersonal conflicts at work range from minor disagreements to severe violence. Non-physical violence, however, such as incivility or serious verbal abuse directed at nurses, is more common (Spector et al., 2014). Acts of physical violence are most prevalent in psychiatric, geriatric and emergency departments, and are committed by patients in about two-thirds of cases, and by patients’ family or friends in about one third. Approximately 10% of violent acts are committed by other nurses, physicians, or staff. The results from Polish studies are alarming (Merecz et al., 2006, 2009). A majority of nurses had experienced aggression in the form of raised voices and threats, and had experienced a dangerous attitude or vulgar behavior in the presence of co-workers and in the presence of patients (Kowalczuk et al., 2011). Interpersonal conflicts at work are a common stressor and they may be taken home (Spector et al., 2014). Therefore, our study examines the role that conflicts at work and the weight of workload may play in turnover intentions.

### Work Engagement Promotes Investment and Protects Resources

Work engagement is an indicator of a personal (energetic) resource that is brought into an organization by the employees. Time spent working may be sometimes stressful, but it engages people and makes their working life meaningful. Work engagement is defined as “a positive, fulfilling work-related state of mind that is characterized by vigor, dedication, and absorption” (Schaufeli et al., 2002, p. 74). It is one of the most important components of subjective well-being at work (Bakker and Oerlemans, 2011). Vigor is reflected in high levels of energy and cognitive resilience while at work, the willingness to invest effort at work, and perseverance in the face of difficult situations at work. Dedication is reflected in an experience of a sense of significance, enthusiasm, inspiration, pride, and challenge while at work. Finally, absorption is reflected in the state of being fully focused and deeply engrossed in one’s work, so much that time passes quickly and one has difficulty detaching oneself from the work.

We have assumed that work engagement is an indicator of a personal resource that is important for the employee’s performance at work. A nurse is expected to develop smooth relationships with the patients, to be empathetic, and thus engaged. Work engagement is positively correlated with intrinsic motivation (Schaufeli and Salanova, 2007). An engaged employee is energetic, effective in work activities, and experiences himself or herself as able to deal well with work-related demands (Schaufeli et al., 2002). Employees who have high levels of work engagement are more committed to delivering high-quality performance at work, receive higher work ratings from their supervisors, are promoted more rapidly within the organization, and enjoy higher levels of gratitude from their customers (Schaufeli et al., 2001). An engaged employee is also willing to carry out both in-role behavior and extra-role behavior at work (Bakker and Schaufeli, 2008). Thus, according to past research, it may be expected that at least one aspect of engagement, vigor, is negatively correlated with turnover intentions. Organizations can transform the engagement of employees as an organizational resource to build a competitive advantage. We suggest that different levels of work engagement indicate different degrees of investment of personal resources in the work process.

### Turnover Intentions as Consequences of Threat of Loss of Resources

Employees adopt various strategies to manage stressors between work and home. One of these strategies is to develop a turnover intention, that is, considering leaving one’s job. We define two turnover intentions: (a) the intention to leave the present workplace; and (b) the intention to leave the nursing profession. Turnover intentions are high in nurses throughout the world (e.g., Chan et al., 2008; Binkowska-Bury et al., 2010; Sawatzky and Enns, 2012; Homburg et al., 2013). The reason for turnover intentions is not only related to nurses’ dissatisfaction with pay and benefits, as is true in Macao nurses (Chan et al., 2008). In Netherlands, it is also determined by the nurses’ general dissatisfaction with management and leadership quality, with their inability and lack of possibilities to manage WFC/FWC, but not by career development opportunities (Homburg et al., 2013), while in Japan it is determined by unfulfillment of the psychological contract and to a lack of opportunity for advancement (Takase et al., 2016).

It should be noted that FWC has been found to be directly and indirectly related to turnover intentions in employees in a financial firm (Frone et al., 1997), school teachers and administrators, real estate sales personnel, small-business owners (Netemeyer et al., 1996), police department employees (MacEwen and Barling, 1994), government employees (Brotheridge and Lee, 2005), and bank employees (Hammer et al., 2003). Amstad et al. (2011) have shown that both WFC and FWC are positively correlated with turnover intention. Chen et al. (2015) showed that among Taiwanese nurses, WFC mediates the relationship between job satisfaction and turnover intention to leave the present organization. In addition, one’s level of engagement is the key predictor of turnover intentions in Canadian registered nurses’ working at emergency departments (Sawatzky and Enns, 2012).

Work-family conflict and FWC have been found to be moderately correlated, but their antecedents and consequences differ. Nohe et al. (2015) showed that WFC has a stronger effect than FWC on work-specific responses to demands such as burnout, depression, and psychosomatic symptoms. Byron’s (2005) meta-analysis showed that WFC is related to the perceived intensity of occupational and family related stress. Mesmer-Magnus and Viswesvaran’s (2005) meta-analysis showed that WFC has a moderate correlation with work overload and role conflict, as well as low correlations with many family related stressors (e.g., perceived family demand, parental demands, within-family personal conflict, time-commitment to family). In contrast, in a sample of Polish nurses (Baka, 2013), FWC had almost zero correlations with age, job seniority, and working hours per week. The only significant positive correlation (0.19) was between FWC and the number of children. The number of hours devoted to professional work was positively related to WFC in Baka’s study. A similar pattern of correlations was shown in a convenience subsample of Polish women (Lubranska, 2014, **Table 3**).

Previous research has indicated that WFC is stronger than FWC (Grandey and Cropanzano, 1999; Netemeyer et al., 2005; Baka, 2013; Lubranska, 2014), that is, a negative impact of work on family life occurs more often than the reverse (Geurts et al., 2003).

In summary, according to COR theory, those who experience a high WFC feel high work stress because resources are lost in the process of combining work and family roles. Those who experience a high FWC feel that family duties compete for resources that are needed to fulfill their professional role. FWC may also interfere with work-related performance, but this aspect has rarely been investigated.

### Rationale and Aims

The role of WFC/FWC with job demands and work engagement in turnover intentions in nurses is not well-investigated in post-transformational countries. We therefore aimed to investigate, using COR theory, the relationship between these variables in registered nurses in Poland.

Drawing on COR theory as our theoretical framework, we believe that: (1) Excessive demands harm personal resources, contributing to loss of resources; (2) WFC and FWC constitute a struggle for resources between two important domains of life, because if work demands are too high and one has to reconcile them with household chores, then one has to give something up, which leads to a threat of loss of resources; (3) Engagement is an investment of resources – to the benefit of patients but not necessarily leading to any reward for nurses; however, it may improve relationships with patients and in return build new resources for nurses (respect and recognition, improvement of professional, and social skills). Others have argued that conflict between one’s professional and family lives may lead to exhaustion and burnout (e.g., Leineweber et al., 2014).

The present study focuses on turnover intentions, which is a current world-wide problem (Chan et al., 2008; Binkowska-Bury et al., 2010; Homburg et al., 2013). Using a cross-sectional design, we examine, in the framework of this theory, nurses’ perceptions of job demands, work-family interface and work engagement, and to which extent these variables are associated with turnover intentions.

The aims of our study are twofold. Firstly, we examine the extent to which perceived job demands (workload and interpersonal conflicts at work) and engagement (vigor, dedication, and absorption) are associated with turnover intentions (the intention to leave the present workplace and the intention to leave the nursing profession). Secondly, we attempt to determine whether levels of WFC and FWC moderate these associations. Studies that seek to illuminate the role of such factors for the turnover intentions of Polish nurses have not previously been published.

## Materials and Methods

### Rationale for Recruitment of Participants and Procedure

We were interested in examining a group that can truly experience WFC. We therefore aimed to focus explicitly on nurses who had additional duties at home and a responsibility for a family. Female nurses, employed in various hospitals and public or private clinics in southern Poland, and who were taking a continuing education course, required for registered nurses in the European Union, were invited to take part. They were not offered any reward for their participation in our study. The study was conducted in accordance with applicable ethical rules (the Helsinki Declaration) and was reviewed by the local ethics committee (DNR 2014/730 B 22, University West).

Those who accepted the invitation to take part in the study were informed in writing that participation in the study would be voluntary and anonymous and the data would be used for research only. They gave their written consent to participation in the study. Questionnaires were completed by the participants during the free time between lectures. Two-hundred and two questionnaires were returned. Data from 14 nurses, who did not fulfill the inclusion criterion of living with a partner and/or having children, were excluded. Data from the remaining 188 female nurses (mean age, *M* = 41 years, *SD* = 5.5, range: 28–56) were analyzed. Their average duration of employment was 19.5 years (*SD* = 7, range: 1–35 years). Around 70% of the participants had at least two children, which is a circumstance that may intensify WFC. **Table 1** presents descriptive statistics for the participants.

**Table 1:** Individual and organizational characteristics of the nurses (*N* = 188).

Variable	*n*	%	Missing data
Has a partner	178	95	0
Has an employed partner	166	94	2
Has no children	9	5	0
1 child	49	26	
2 children	82	44	
3–5 children	48	25	
Supervises others	20	11	21
Works at a hospital^a^	120	64	33
Works at a public institution^b^	119	63	15
In full-time employment	168	89	8

*^a^The remaining participants worked at public or private clinics. ^b^The remaining participants worked at a non-public or a private institution.*

### Measures

**Quantitative workload** was assessed by Spector and Jex’s (1998) five-items Quantitative Workload Inventory (QWI). The Polish version was used (Baka and Cieslak, 2010). Responses are given on a scale of 1 (*Less than once per month or never*) to 5 (*Several times per day*). An example item is “How often does your job require you to work very fast?” The sum of scores of the five-items indicates the quantity of work in the participant’s work situation. High scores correspond to a high workload. In the current study Cronbach’s alpha was 0.80.

**Interpersonal conflicts at work** were measured by Spector and Jex’s (1998) four-items Interpersonal Conflict at Work Scale (ICAWS). The Polish version was used (Baka and Cieslak, 2010). Participants are asked to indicate on a five-point scale how often each of the four events described by the statements occurs at work. The response options range from 1 (*Less than once per month or never*) to 5 (*Several times per day*). An example item is “How often do you get into an argument with others at work?” High scores correspond to frequent conflicts with others, and describe how often the respondent experiences disagreements or is poorly treated at work. In our study, Cronbach’s alpha was 0.61.

**Work engagement** was measured using the Polish version of the Utrecht Work Engagement Scale (UWES-9; Schaufeli and Bakker, 2003). The UWES-9 comprises nine statements that measure the concepts of vigor (“At my work, I feel that I am bursting with energy”), dedication (“I am enthusiastic about my job”), and absorption (“I feel happy when I am working intensely”). Each item is rated on a seven-point scale that ranges from 0 (*Never*) to 6 (*Always/every day*). The average of the sum of scores divided by the number of items gives the level of engagement. Higher scores indicate a higher work engagement. Cronbach’s alpha in the present group was 0.73, 0.73, and 0.64 for vigor, dedication, and absorption, respectively.

**Work-family conflict** and family work conflict were measured by the Polish versions of the Work-Family Conflict and Family Work Conflict Scales (Netemeyer et al., 1996; Zalewska, 2008). These scales assess how work affects family life and *vice versa*. Each scale comprises five-items. The scale ranges from 1 (*Strongly agree*) to 7 (*Strongly disagree*). An example of an item from the WFC scale is “The amount of time my job takes up makes it difficult to fulfill family responsibilities.” The items of FWC parallel the items of WFC, reversing the source of the stressor. Cronbach’s alpha in the present study was 0.89 for WFC and 0.88 for FWC.

**Turnover intentions** were assessed by two single-item scales. **Intention to leave the present workplace** was measured by the following item: “During the recent period I have considered leaving this hospital/organization to begin work in another hospital/organization,” while **Intention to leave the nursing profession** was assessed by the item “During the recent period I have considered leaving the nursing profession.” Each item is rated on a four-point scale that ranges from 0 (*Never*) to 4 (*Always*). High scores indicate high levels of turnover intentions.

#### Control Variables

We asked nurses about their individual and family characteristics in order to describe the group. Some of these variables were chosen as control variables in our analyses, because these may denote degrees of family related duties and thus may relate to WFC/FWC and to turnover intentions. Those included nurses’ age, number of children, living with a partner (coding 0 *without a partner*; 1 *with a partner*) and partner employment status (coding 0 *an unemployed partner*; 1 *an employed partner*).

### Treatment of Data and Statistical Analyses

Standard descriptive statistics were used to summarize the data. Exact *p*-values for both significant and non-significant results, and bootstrap corrected, 95% bias corrected and accelerated confidence intervals (BCa CIs) around the means estimated for 1000 samples, were reported. Analyses were performed with SPSS version 23. The distributions of the variables were checked for severe deviations from normality. Non-parametric (Spearman’s Rho, Kendall’s tau) and Pearson correlation coefficients were calculated. The Bonferroni correction was applied to the significance tests.

**FIGURE 1:**
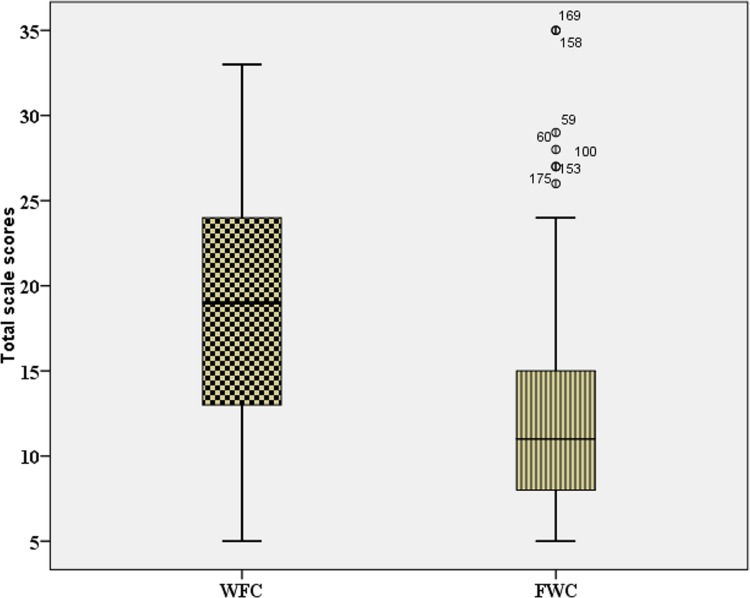
**The distribution of scores of work-family conflict (WFC) and family work conflict (FWC) for the group of Polish nurses (*N* = 188).** The figure shows the minimum and maximum values, the medians (the dark lines inside the boxes), the lower quartiles (the bottom lines in the boxes), the upper quartiles (the top lines in the boxes), and extreme values (outliers) of the FWC.

The distribution of FWC was positively skewed (see **Figure 1**), and we therefore report median values of WFC and FWC. We compare values for these variables using a non-parametric test, the Wilcoxon signed-rank test. The effect size estimate was calculated using a *z*-score (see Field, 2013, p. 227).

We examined the effect on the dependent variables of being below or above the median in WFC and FWC. Those with median scores were not included in these analyses. Differences in the continuous variables between low and high groups were examined by independent *t*-test, and Levene’s test was used to examine whether the two groups had equal variances. We computed effect sizes (Cohen’s *d*) (Cohen, 1988) of the differences in means using an on-line calculator^1^. Differences in frequencies of two control categorical variables (has a partner, partner employed) were examined by Chi-squared tests. To assess the significance of the difference between two correlation coefficients, *r*_a_ and *r*_b_, found in two independent samples, we calculated values of *z* using the Fisher *r*-to-*z* transformations through an online calculator^2^.

We carried out two hierarchical multiple analyses in order to determine the relative importance of the measures examined for turnover intentions. Three control variables were included as a first step. In order to generalize our results to different groups of nurses we cross-validate the model by reporting two types of adjusted *R*^2^; one computed by SPSS using Wherry’s equation (Wherry, 1931), and another one, computed by hand, using Stein’s formula (see Field, 2013, p. 312). The latter calculation was done because Wherry’s equation has been criticized due to a poor ability to predict how well our regression model “would predict scores of a different sample of data from the same population” (Field, 2013, p. 312).

## Results

### Descriptive Statistics

Work-family conflict was positively correlated with FWC (**Table 2**). This relationship was still significant after the Bonferroni correction, and the effect size was moderate.

**Table 2:** Correlations and descriptive statistics of the variables for the participants (*N* = 188).

Variable	*M*	*SD*	*S*	*K*	1	2	3	4	5	6	7	8
(1) QW	19.17	3.81	-0.46	-0.28								
(2) ICAWS	7.33	2.85	0.91	1.05	0.07							
(3) VI	3.77	0.97	-0.12	-0.58	-0.09	-0.19						
(4) DE	4.51	0.98	-0.41	-0.29	0.03	-0.19	0.66*					
(5) AB	3.04	1.25	0.25	-0.75	0.18	-0.25*	0.21	0.29*				
(6) ILW	2.03	0.79	0.41	-0.27	0.26*	0.45*	-0.37*	-0.34*	-0.03			
(7) ILNP	1.52	0.67	0.93	-0.29	0.23	0.50*	-0.32*	-0.27*	-0.06	0.54*		
(8) WFC	18.45	6.53	-0.08	-0.81	0.38*	0.21	-0.25*	-0.14	0.09	0.35*	0.31*	
(9) FWC	11.96	5.48	1.42	3.19	0.12	0.11	-0.05	0.04	0.45*	0.14	0.12	0.31*

*p < 0.05, after the Bonferroni correction (0.05/36 = 0.0014). *S*, skewness; *K*, kurtosis; QW, quantitative workload; ICAWS, interpersonal conflicts at work; VI, vigor; DE, dedication; AB, absorption; ILW, intention to leave the present workplace; ILNP, intention to leave the nursing profession; WFC, work-family conflict; FWC, family work conflict. Results are based on 1000 bootstrap samples (BCa CIs are not shown).

Results in **Table 2** show also that WFC and FWC were positively related to job demands and to turnover intentions, and that WFC and FWC were negatively related to the components of engagement. Few correlations remained statistically significant after the Bonferroni correction. WFC was positively related to workload and to turnover intentions, and was negatively related to vigor. The strengths of these correlations, and thus, the effect sizes, were moderate. In contrast, FWC was positively related only to absorption, and this effect size was moderate.

The relative strength of WFC and FWC was examined by the related samples Wilcoxon signed rank test. WFC levels were significantly higher (median = 19) than FWC levels (median = 11), *T* = 959, *p* < 0.001, *r* = -0.72. The effect size of -0.72 corresponds to a large difference in the levels of these two measures (above Cohen’s benchmark of 0.50). It is apparent that WFC was experienced with greater average intensity than FWC. **Figure 1** illustrates the distribution of the two variables.

### To What Extent are Perceived Job Demands and Engagement Associated with Turnover Intentions?

**Table 2** shows that the mean value of the intention to leave the present organization was larger than it was for the intention to leave the nursing profession. We compared these values using a paired *t*-test. The difference, 0.51, BCa 95% CI [0.41, 0.61], was significant *t*(187) = 9.89, *p* < 0.001.

We performed two separate hierarchical multiple regression analyses in order to examine the extent to which perceived job demands (workload and interpersonal conflicts at work) and engagement (vigor, dedication, and absorption) are associated with turnover intentions (the intention to leave the present workplace and the intention to leave the nursing profession). The control variables relevant for WFC (age, the number of children, has an employed partner) were introduced in step 1, and the variables related to personal resources (job demands, WFC, work engagement) were entered in step 2. The correlations of turnover intentions with FWC and absorption were weak (0.14, 0.12 and -0.03, -0.06, respectively, see **Table 2**), and despite the fact that past research has suggested that FWC predicts turnover intention (e.g., Brotheridge and Lee, 2005) we excluded FWC, as well as absorption, from the analyses.

**Table 3** illustrates a summary of the regression model of antecedents of nurses’ intention to leave the present workplace. The control variables had no impact: *R*^2^ was significantly different from zero only at the end of the second step. The adjusted *R*^2^ value of 0.37 indicates that more than a third of the variability in nurses’ turnover intention to leave the present workplace is predicted by WFC, job demands (interpersonal conflicts at work, quantitative workload) and engagement (vigor and dedication). This regression model indicates that a high level of interpersonal conflicts at work contributes the most to that prediction while high quantitative workload and low vigor contributed modestly. Neither WFC nor dedication add anything to the prediction.

**Table 3:** Linear model of antecedents of an intention to leave the present workplace, with 95% Bias corrected and accelerated (BCa) confidence intervals (CIs) in parentheses, among Polish nurses (*N* = 188).

Measure	*B* [BCa 95% CI]	*SE B*	β	*p*
**Step 1**				
Constant	3.07 [1.736, 4.372]	0.67		<0.001
Age	-0.02 [-0.037, 0.005]	0.01	-0.11	0.141
No of children	-0.05 [-0.172, 0.061]	0.06	-0.07	0.379
Has an employed partner	-0.14 [-0.581, 0.345]	0.24	-0.04	0.558
**Step 1**				
Constant	1.90 [0.404, 3.233]	0.68		0.006
Age	-0.01 [-0.024, 0.012]	0.01	-0.05	0.496
No of children	-0.01 [-0.113, 0.093]	0.06	-0.01	0.874
Has an employed partner	-0.07 [-0.368, 0.244]	0.15	-0.02	0.590
Work-family conflict	0.02 [-0.002, 0.039]	0.01	0.15	0.067
Interpersonal conflicts at work	0.09 [0.059, 0.123]	0.02	0.34	0.001
Quantitative workload	0.04 [0.004, 0.069]	0.02	0.17	0.030
Vigor	-0.18 [-0.310, -0.036]	0.02	-0.22	0.014
Dedication	-0.11 [-0.262, 0.038]	0.07	-0.14	0.132

*R^*2*^ = 0.02, adj*R*^*2*^ = 0.00 for step 1 (*p* = 0.386); Δ*R*^*2*^ = 0.38, Adj*R*^2^ = 0.37 (using Wherry’s equation), and 0.32 (using Stein’s equation, see Field, 2013, p. 312) for step 2 (*p* < 0.001). *B*, unstandardized coefficient; *SE*, standard error; β, standardized coefficient. CIs and *SE*s are based on 1000 bootstrap samples.*

**Table 4** presents a summary of the regression model of antecedents to nurses’ intention to leave the nursing profession. Also in this model, *R*^2^ was significantly different from zero only at the end of the second step, which means that control variables had no impact on this prediction. The adjusted *R*^2^ value of 0.34 indicates that about a third of the variability in nurses’ turnover intention to leave the nursing profession is explained by WFC, job demands (interpersonal conflicts at work, quantitative workload) and engagement (vigor and dedication). This regression model indicates that a high level of interpersonal conflicts at work contributes the most to that prediction, while high quantitative workload and low vigor contributed modesty. Neither WFC nor dedication add anything to the prediction.

**Table 4:** Linear model of antecedents of an intention to leave the nursing profession, with 95% BCa CIs in parentheses, among Polish nurses (*N* = 188).

Measure	*B* [BCa 95% CI]	*SE B*	β	*p*
**Step 1**				
Constant	2.26 [0.884, 3.413]	0.69		0.002
Age	-0.01 [-0.028, 0.015]	0.01	-0.05	0.500
No of children	-0.04 [-0.133, 0.050]	0.47	-0.06	0.390
Has an employed partner	-0.20 [-0.781, 0.362]	0.76	-0.07	0.469
**Step 2**				
Constant	0.90 [-0.534, 2.117]	0.72		0.203
Age	0.00 [-0.011, 0.018]	0.01	0.03	0.649
No of children	0.00 [-0.079, 0.087]	0.04	0.01	0.918
Has an employed partner	-0.12 [-0.609, 0.350]	0.21	-0.04	0.546
Work-family conflict	0.01 [-0.007, 0.024]	0.01	0.09	0.265
Interpersonal conflicts at work	0.10 [0.074, 0.123]	0.01	0.43	0.001
Quantitative workload	0.03 [0.001, 0.055]	0.01	0.16	0.024
Vigor	-0.14 [-0.267, -0.010]	0.06	-0.21	0.021
Dedication	-0.04 [-0.161, 0.088]	0.06	-0.06	0.512

*R^*2*^ = 0.01, adj*R*^*2*^ = -0.01 for step 1 (*p* = 0.646); Δ*R*^*2*^ = 0.36, Adj*R*^*2*^ = 0.34 (using Wherry’s equation), and 0.29 (using Stein’s equation, see Field, 2013, p. 312) for step 2 (*p* < 0.001). *B*, unstandardized coefficient; *SE*, standard error; β, standardized coefficient. CIs and *SE*s are based on 1000 bootstrap samples.*

The standardized regression coefficients (β), displaying the relative importance among all examined predictors in each regression analysis, indicate that the variable “Interpersonal conflicts at work” was the most important for turnover intentions.

### Comparison between Nurses with Low and High Work-Family Conflict

There were no significant differences between those with low and high WFC in any of the examined control variables (data not shown). For example, the number of children was similar in those with low WFC (*M* = 1.95, *SD* = 1.07) compared to those with high WFC (*M* = 1.99, *SD* = 0.87). **Table 5** shows that participants with high WFC experienced significantly more job demands (quantitative workload and interpersonal conflicts) and significantly less engagement (vigor, dedication, and absorption) and greater turnover intentions than participants with low WFC. The effect sizes of the differences in means were large for both turnover intentions. The level (low vs. high) of WFC had non-significant effects on associations between turnover intentions and the examined variables.

**Table 5:** Comparisons between nurses with low and high values of work-family conflict (WFC).

Variable	Low WFC (*n* = 92)		High WFC (*n* = 86)		*t*	*df*	*p*	*d*
	*M*	*SD*	*M*	*SD*				
Intention to leave the present workplace (ILW)	1.75	0.69	2.31	0.77	5.16	176	<0.001	0.80
Intention to leave the nursing profession (ILNP)	1.34	0.54	1.71	0.73	3.79	156	<0.001	0.58
FWC	10.52	3.99	13.34	6.36	3.51	141	0.001	0.53
Quantitative workload	18.14	3.67	20.27	3.62	3.87	176	<0.001	0.58
Interpersonal conflicts	6.68	2.28	7.99	3.21	3.13	153	0.002	0.47
Vigor	4.04	0.93	3.49	0.94	3.97	176	<0.001	0.59
Dedication	4.75	0.93	4.27	0.99	3.36	176	0.001	0.50
Absorption	2.93	1.20	3.09	1.26	0.86	176	0.390	0.13

**Correlations with ILW**	***r*_LowWFC_**	***p***	***r*_HighWFC_**	***p***		***z***	***p***	

Quantitative workload	0.12	0.123	0.26	0.014		0.95	0.342	
Interpersonal conflicts	0.47	<0.001	0.37	<0.001		0.80	0.424	
Vigor	-0.28	0.007	-0.36	0.001		0.58	0.562	
Dedication	-0.30	0.004	-0.24	0.030		0.42	0.675	
Absorption	-0.05	0.648	-0.01	0.903		0.26	0.795	

**Correlations with ILNP**								

Quantitative workload	0.18	0.084	0.17	0.125		0.07	0.944	
Interpersonal conflicts	0.49	<0.001	0.47	<0.001		0.17	0.865	
Vigor	-0.29	0.005	-0.23	0.030		0.42	0.675	
Dedication	-0.29	0.005	-0.17	0.110		0.83	0.407	
Absorption	-0.14	0.176	-0.03	0.776		0.73	0.465	

*WFC, work-family conflict; FWC, family work conflict. The group with low WFC comprised participants with a value of WFC below the median of 19, while the group with high WFC comprised those with a value of WFC above this level. Participants (*n* = 10) with WFC = 19 were excluded from the analyses. *p* is two-tailed. Results are based on 1000 bootstrap samples (BCa CIs are not shown).*

### Comparisons between Nurses with Low and High Family Work Conflict

There were no significant differences between participants with low/high WFC in any of the examined control variables (data not shown). Similarly as for WFC, the number of children was similar in those with low FWC (*M* = 2.00, *SD* = 1.04) compared with those with high FWC (*M* = 1.95, *SD* = 0.99). As is shown in **Table 6**, nurses with high FWC experience higher WFC than those with low FWC. The effect size of the difference was moderate. It should be noted that participants with high FWC had a significantly higher level of absorption. This effect size was large. Participants who experienced a high negative impact of family on work (those with high FWC) experienced also a higher frequency of interpersonal conflict at work and tended to want to leave the nursing profession. The effect sizes of the differences in means were small for both turnover intentions. The level (low vs. high) of FWC had non-significant effects on associations between turnover intentions and examined variables.

**Table 6:** Comparisons between nurses with low and high values of family work conflict.

Variable	Low FWC (*n* = 90)		High FWC (*n* = 84)		*t*	*df*	*p*	*d*

	***M***	***SD***	***M***	***SD***				
Intention to leave the present workplace (ILW)	1.92	0.80	2.11	0.79	-1.52	172	0.130	0.24
Intention to leave the nursing profession (ILNP)	1.40	0.63	1.66	0.70	-2.57	172	0.011	0.39
WFC	16.22	7.08	20.39	5.14	4.47	162	<0.001	0.67
Quantitative workload	18.98	4.10	19.54	3.43	-0.98	172	0.328	0.15
Interpersonal conflicts	6.86	2.75	7.76	2.95	-2.10	172	0.037	0.32
Vigor	3.89	0.94	3.65	1.00	1.56	172	0.120	0.25
Dedication	4.66	1.02	4.40	0.95	1.73	172	0.085	0.26
Absorption	2.66	1.33	3.52	0.04	-4.76	167	<0.001	0.91

**Correlations with ILW**	***r*_LowFWC_**	***p***	***r*_HighFWC_**	***p***		***z***	***p***	

Quantitative workload	0.40	<0.001	0.17	0.113		1.63	0.103	
Interpersonal conflicts	0.46	<0.001	0.45	<0.001		0.08	0.936	
Vigor	-0.43	<0.001	-0.31	0.004		0.90	0.368	
Dedication	-0.38	<0.001	-0.24	0.028		1.01	0.313	
Absorption	-0.15	0.160	0.08	0.475		0.46	0.646	

**Correlations with ILNP**								

Quantitative workload	0.32	0.002	0.14	0.222		1.24	0.215	
Interpersonal conflicts	0.47	<0.001	0.55	<0.001		0.70	0.484	
Vigor	-0.36	0.001	-0.29	0.007		0.51	0.610	
Dedication	-0.33	0.002	-0.18	0.096		1.04	0.298	
Absorption	-0.22	0.038	-0.04	0.750		1.19	0.234	

*WFC, work-family conflict; FWC, family work conflict. The group with a low FWC comprised participants with a value of FWC below the median of 11, while the group with a high FWC comprised those with a value of FWC above this level. Participants (*n* = 14) with FWC = 11 were excluded from the analyses. *p* is two-tailed. Results are based on 1000 bootstrap samples (BCa CIs are not shown).*

## Discussion

This study has examined the relationships between WFC, FWC, and relevant work-related variables in a group of nurses from Poland, a post-transformation country with a long tradition of high full-time employment rates for women (Matysiak and Steinmetz, 2008). It is known that an increase in work demands occurs over time in post-transformation countries, because of rapid changes in society, increased competition, and thus, more demanding work conditions. In summary, in our results, WFC and FWC were moderately correlated. Mean WFC was higher than mean FWC. WFC was positively related to quantitative workload and turnover intentions, and negatively related to vigor. In contrast, FWC was positively related only to absorption. Next, turnover intentions were predicted by high interpersonal conflicts at work, high quantitative workload, and low vigor. Further, nurses with high WFC (a negative impact of work on family life) experienced greater job-related demands (quantitative workload and interpersonal conflicts at work) and less work engagement (vigor, dedication) than nurses with low WFC (moderate effect sizes). In addition, they declared greater turnover intentions (large effect size). Finally, nurses with high FWC (a negative impact of family life on work) experienced higher levels of interpersonal conflicts at work and declared an intention to leave the nursing profession (small effect sizes), felt higher WFC, and had a higher level of absorption (moderate and large effect size, respectively) than those with low FWC. However, levels of WFC and FWC did not significantly moderate these associations.

The magnitude of WFC was greater than that of FWC in our study (**Table 2**), as has previously been shown in other countries (e.g., Grandey and Cropanzano, 1999), as well as in a convenience sample of Polish people (e.g., Lubranska, 2014). This was previously also shown in Polish nurses (Baka, 2013). This result is here confirmed in a new group of Polish nurses. It should be noted that mean values of WFC and FWC were higher in both samples of Polish nurses compared with Lubranska’s sample, and that these values were greater in Baka’s study than in the current study. We cannot state that the nurses in our study manage their resources more economically than those in Baka’s study.

Work-family conflict was positively correlated (with a moderate effect size) with job demands, quantitative workload, and interpersonal conflicts. An excessive workload is one kind of work demand that is linked to WFC (Geurts et al., 2003). Further, the occurrence of interpersonal conflicts, which is an example of an emotional demand, is also linked to WFC (Peeters et al., 2005). WFC, but not FWC, had a positive correlation (moderate effect size) with turnover intentions, which is theoretically and empirically reasonable (Nohe and Sonntag, 2014; Chen et al., 2015). This result is not in line with the result of Grandey and Cropanzano (1999), which may be partly explained by the fact that WFC and intention to leave the current job were assessed by other measures in the latter study. The strength of the relationship between FWC and one of the dimensions of engagement, absorption, was moderate. It is possible that absorption is related to an excessive commitment to work, and thus, excess investment of resources without gain. Nurses who are more absorbed by their work may feel discomfort when household responsibilities detach them from their beloved work.

The current study showed that high levels of interpersonal conflicts and high quantitative workload and low vigor predicted both an intention to leave the present workplace and an intention to leave the nursing profession. So a common important feature for the two types of turnover intentions is high job demands and a low level of vigor. Job demands deplete personal resources, especially energetic resources. Vigor represents additional energetic resources that can be invested, but in our study the nurses had no more energy to invest, neither in their present workplace nor in the nursing profession. In fact, they could only offer negative energy, their exhaustion, because about 20% of the variation in both turnover intentions was explained by poor vigor. Thus the relationship between interpersonal conflicts, quantitative workload and vigor seems to demonstrate competition for resources in two important domains: family and work. Low vigor predicted both types of turnover intentions, which is in line with the concept of loss spirals in COR theory (Hobfoll, 1989), as loss spirals develop when nurses lack energy to compensate for poor organizational resources in the form of work conditions. Miserable work conditions may kill the internal energy and willingness to invest effort in nurses’ work activity. Employees who feel great vigor at work are highly motivated by their jobs and are likely to remain persistent when encountering difficulties (Mauno et al., 2006). In contrast, our nurses showed low willingness to invest effort in their work activity and did not show perseverance when faced with difficulties. It may be assumed that these nurses believe in better working conditions in a new workplace. Turnover is a strategy of managing the balance between work and family. The current trend toward globalization in the nursing profession, which provides good opportunities to leave the present workplace for another that may offer better working conditions, may improve the retention of nurses in their profession. It is, however, not a good solution for countries whose populations are aging and where many nurses are needed.

In our study, nurses with high scores for WFC and FWC differed from those with low scores in the degree of interpersonal conflicts at work. FWC was, however, more intense in nurses with high WFC. These results (see **Table 5**) are compatible with the COR theory, which predicts that personal resources (here: vigor and dedication) will deteriorate when excessive demands (here: quantitative workload and interpersonal conflicts at work) are experienced, like in our nurses with high WFC. In addition, these nurses tended to declare both an intention to leave the present workplace and an intention to leave the nursing profession.

In past research on Polish nurses (Baka, 2013), family size was found to intensify WFC, because nurses with many children may require more time and energy to invest in the children’s school and free-time activities compared with nurses without children or with only one child, and these conditions are more manageable when one’s partner is not employed. In the current study, however, family size (here, the number of children) or other traditional covariates in WFC research, such as an unemployed partner, were not significantly associated with either WFC or FWC, or with turnover intentions.

Our results are consistent with the COR theory, which states that when personal resources are depleted, people will conserve their remaining resources by intending to relieve the stress. Nurses’ personal resources may be depleted and their self-esteem as professional nurses and respectable human beings may be threatened. One of the protesters on 24 May 2016, a nurse with 35 years of experience, said: “There are too few of us. But, unfortunately, we can’t clone ourselves.” This is a situation that is risky for patients’ safety and the standard of work in the nursing profession.

The current study has its methodological shortcomings, which should be addressed. First, all studies on WFC/FWC are based on self-reports, which may increase the risk of common-method variance. Future research on the relationship between WFC/FWC and other work-related variables should comprise some objective indicators as alternative or additional measures. Second, the cross-sectional research design limits the possibility of drawing conclusions about the causal nature of the relationships and gives us no idea of changes in behavior and perceptions over time. Therefore, future studies that aim at explaining relationships between WFC/FWC, job demands, and engagement and turnover intentions in nurses may, preferably, have longitudinal/processual designs which gives us the possibility to employ path analyses. Third, Cronbach’s alpha for absorption was relatively low in the present sample of nurses. A similar figure, however, was found in a large sample of Spanish nurses (Garrosa et al., 2011), and it should be remembered that this measure comprises only three-items. Fourth, the measure used to assess interpersonal conflicts suffers from limitations. Several recent studies (such as Sliter et al., 2014) have used modified versions of the scale, and asked respondents about different sources of work conflict. Fifth, turnover intentions have been operationalized with single-item measures that may be unreliable (Voydanoff et al., 1988; Rice et al., 1992). We argue, however, that in this case assessment by a single question is appropriate, and single-item measures of turnover intention have been successfully used in earlier studies (see, for example, Parasuraman, 1982). Finally, we have not considered links between non-work related variables and FWC. For example, we have not collected data concerning the number of children living at home (Kinnunen and Mauno, 1998). We assessed only the number of children, which had no significant associations with the studied variables. Examples of other demands that we have not examined are home workload (Peeters et al., 2005), caring for sick or elderly parents (Marks, 1998), marital conflicts (Fincham, 2003), family climate (Michel et al., 2011), and leader support (Nohe and Sonntag, 2014). In the future, a dyadic design, in which data regarding WFC/FWC are collected among the nurses’ family members, could be considered. We had no opportunity to interview the nurses’ family members and ask them how they experienced the nurses’ work. We have at least gone one step further than those who only ask if their participants are married.

To which populations may our results be generalized? Our study was not planned to have a sample that possesses all the characteristics of the population in similar proportions. However, our nurses represent a broad range of experienced nurses with a mean age (41 years) similar to the mean age of Polish nurses (45 years). We therefore believe that generalization of our results is possible to Polish nurses in general. We can probably not generalize our results to male nurses, nor to nurses who have neither a partner nor children, but it should be remembered that 96% of Polish nurses are women, and the majority of them have a partner and/or children. Because our study concerns a new application of the concepts of COR theory, we have no similar results to compare with.

We argue that a poor work environment causes personal resources to acquire a different meaning for the employees than in more optimal conditions (Fischer and Boer, 2011; Xanthopoulou et al., 2012). Our results can possibly be applied to nurses from other countries, working under similar (poor) working conditions as those that participated in our study.

This is the first study that has examined how a constellation of WFC/FWC, job demands, and work engagement may predict turnover intentions of Polish nurses, based on COR theory. Future research should include additional measures (e.g., burnout, self-esteem, personality), and the present study may give some guidelines for which measures should be included in the regression model. Further, processual models are needed to investigate relationships between job characteristics that are related to the management of resources, as well as dyadic studies in the family or private domain. The integration of our results has been based on the group or phenomenon level, a more or less implicit assumption being that all nurses with a high WFC experience intense work demands, and experience these as a threat to their highly valued resources. The COR theory states that an individual’s perception of what constitutes resources and what constitutes threats to these resources is strongly influenced by his or her values, which can, in turn, be regarded as the basis of culture (Hobfoll, 1989).

Some practical implications could be emphasized. Excessive workload and frequent interpersonal conflicts at work, as well as a lower degree of engagement, may lead to a lower efficiency of nurses, which may place the safety of patients at risk. The first practical aim for a manager should be the improvement of working conditions. Organizations need engaged employees not only to build competitive advantage, but also to enable development. This is particularly true in the service sector. The study of White et al. (2014, p. 1634) confirmed that work engagement can be considered to be a personal resource that an employee invests in the organization, and showed that “the engagement of nurses and front-line clinical teams is a major component of creating, developing and sustaining a culture of improvement.” The engagement of nurses thus empowers ward teams to be active and innovative.

Guidance and counseling for nurses in Polish health organizations are required to retain them in the organizations and to promote decent working conditions and a happy life. To our knowledge, the current policy is to scare people not to strike, as this can lead to loss of their jobs, and foster them to believe that it is not possible to improve working conditions because there are no funds for this. Such policies may enhance turnover intentions. We suggest that intervention programs be developed, which should at least deal with the management of work conflicts. Such management is possible by, for example, recruitment and education of managers among experienced nurses and by formulation of guidelines regarding what kind of co-worker behavior is acceptable. In their study of registered nurses, Leineweber et al. (2016, p. 1) concluded that adequate staffing, good leadership, and support for nurses are crucial” for the nurses’ mental health.

We have shown that stress indicators in form of high quantitative workload, interpersonal conflicts at work are significant predictors of turnover intentions. High turnover rates are currently a major problem in post-transformation countries, which is also a global problem. Our results highlight an awareness of the fact that interventions are needed to improve the working conditions of registered nurses, a measure which is in line with others’ (e.g., Leineweber et al., 2016) suggestions for hospital managers to develop “policies and practices to facilitate the successful combination of work with private life for employees” (p. 1).

## Author Contributions

Both authors (AD, BB) substantially contributed to the conceptualization and design of the manuscript, as well as to the acquisition, analysis, and interpretation of data; drafted the manuscript and repeatedly revised and refined it critically for important intellectual content; approved the final version; agreed to be accountable for all aspects of the work in ensuring that questions related to the accuracy or integrity of any part of the work are appropriately investigated and resolved. BB was responsible for the study design, prepared the set of questionnaires and the selection criteria for the sample, and also organized the data collection.

## Conflict of Interest Statement

The authors declare that the research was conducted in the absence of any commercial or financial relationships that could be construed as a potential conflict of interest.

## Footnotes

1 http://www.uccs.edu/~lbecker/

2 http://www.vassarstats.net/rdiff.html
